# Commentary: Facet joint infiltration for chronic low back pain: Is it worthwhile?

**DOI:** 10.4103/0971-3026.45342

**Published:** 2009-02

**Authors:** Ashish Gupta, Sanjiv Sharma

**Affiliations:** Department of Cardiovascular Radiology, All India Institute of Medical Sciences, New Delhi, India

Facet or zygapophyseal joints are innervated by the medial branches of the dorsal rami and are a common source of chronic spinal pain.[1] Based on the criteria established by the International Association for the Study of Pain, facet joints may be a source of chronic pain in 15-45% of patients with chronic low back pain.[2] Facet joint interventions are used to manage chronic facet-mediated spinal pain with a variable efficacy for short-term and long-term relief.[3] Facet joint pain may be managed by intra-articular injections, medial branch blocks, and neurolysis of medial branch nerves. However, conflicting results of the value and efficacy of these different treatment modalities have been reported. It has even been suggested that intra-articular facet joint injections may be no better than placebo for chronic lumbar spine pain.[4] A recent review also found limited evidence for intra-articular injections in the lumbar spine.[5] In contrast, Boswell *et al*,[3] showed moderate evidence for lumbar intra-articular facet joint injections for short-term improvement, but only limited evidence for long-term improvement.

In view of the controversial outcomes of this treatment method, the paper on image-guided lumbar facet joint interventions in this issue of the IJRI assumes significance.[6] Among the 44 selected patients with chronic low back pain treated with facet joint infiltration, 93% patients had pain relief at 1-month and as many as 62% patients sustained this benefit at 6-month follow up, without complication. Despite limitations in study design, small sample size and an in-homogenous patient population, this study provides some evidence for the longer term outcome of this treatment. In facet joint infiltration, a mixture of a local anesthetic and corticosteroid is injected under image guidance with the rationale that the anesthetic will abort the pain spasm cycle and the steroid will reduce inflammation. This can be performed for diagnostic or therapeutic purposes. A recent systematic review of the published literature on this subject commented that the evidence for the effectiveness of intra-articular facet injections is moderate for short- and long-term relief of lumbar facet pain. These authors also concluded that based on best evidence, long-term relief of facet joint pain with medial branch blocks may require local anesthetic injections at intervals of approximately 3-4 months, with or without steroid.[7]

Unfortunately, there is little conformity in the published data in terms of study design, injectable material, outcome measures or follow up data to deduce clear recommendations on the role of this treatment method. An optimal randomized double blind study with a large sample size is warranted to address this issue.
